# Adamantane in Drug Delivery Systems and Surface Recognition

**DOI:** 10.3390/molecules22020297

**Published:** 2017-02-16

**Authors:** Adela Štimac, Marina Šekutor, Kata Mlinarić-Majerski, Leo Frkanec, Ruža Frkanec

**Affiliations:** 1 University of Zagreb, Centre for Research and Knowledge Transfer in Biotechnology, Rockefellerova 10, 10000 Zagreb, Croatia; astimac@unizg.hr; 2 Department of Organic Chemistry and Biochemistry, Ruđer Bošković Institute, Bijenička cesta 54, 10000 Zagreb, Croatia; marina.sekutor@irb.hr (M.Š.); majerski@irb.hr (K.M.-M.); frkanec@irb.hr (L.F.)

**Keywords:** adamantane, targeted drug delivery, liposomes, surface recognition, cyclodextrins, dendrimers

## Abstract

The adamantane moiety is widely applied in design and synthesis of new drug delivery systems and in surface recognition studies. This review focuses on liposomes, cyclodextrins, and dendrimers based on or incorporating adamantane derivatives. Our recent concept of adamantane as an anchor in the lipid bilayer of liposomes has promising applications in the field of targeted drug delivery and surface recognition. The results reported here encourage the development of novel adamantane-based structures and self-assembled supramolecular systems for basic chemical investigations as well as for biomedical application.

## 1. Introduction

**Scheme 1:**
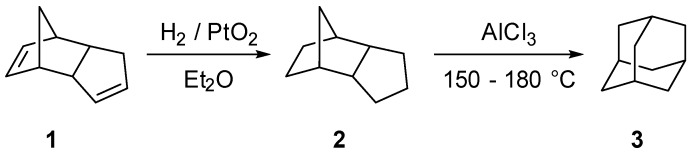
Lewis-acid catalyzed rearrangement leading to adamantane.

Adamantane, a polycyclic cage molecule with high symmetry and remarkable properties [1], is the smallest representative of diamondoids—hydrogen-terminated hydrocarbons with a diamond-like structure [2,3]. It was first isolated in 1933 from crude oil [4], and the first synthesis of adamantane was accomplished in 1941 by Prelog and Seiwerth [5]. However, only after Schleyer discovered the favorable Lewis-acid catalyzed rearrangement procedure leading to the adamantane cage (Scheme 1) [6,7], did adamantane become a widely available scaffold for numerous transformations [8,9]. Studies of biological activity of adamantane derivatives emerged almost parallel to the rapid development of adamantane chemistry. The first adamantane compound applied in medicinal chemistry was amantadine (1-aminoadamantane) that displayed potent anti-Influenza A activity [10,11], marking the beginning of adamantane-based drug discovery [12,13]. The adamantane moiety is nowadays usually introduced into structures of already active drugs in order to increase their lipophilicity and improve their pharmacological properties.

The uniqueness of the adamantyl scaffold for biological application is due to its lipophilicity and ability to ensure drug stability, resulting in enhanced pharmacokinetics of the modified drug candidates [14,15]. The rigid cage moiety protects nearby functional groups from metabolic cleavage and thereby enhances the stability and distribution of the drug in blood plasma [13]. Additionally, the adamantyl moiety can, because of its dimensions and bulkiness, serve as an ideal fit for cavities of various host molecules—e.g., cyclodextrins [16,17]—or act as a blocking agent for cellular ion channels [18,19,20]. Adamantane can also be incorporated into a lipophilic part of the lipid bilayer that constitutes membranes [21], which is an important first step for drug transfer through cell membranes. The mechanisms of these phenomena are still not fully understood and we will here analyze recent studies dealing with these processes.

The focus of this review will be only on adamantyl derivatives used for surface recognition and drug delivery since excellent recent reviews covering the vast area of medicinal application of adamantane derivatives already exist [12,13]. We will therefore give an overview of recent studies dealing with the incorporation of adamantane compounds in liposomes and cyclodextrins and cover the topic of adamantyl dendrimers. We will also provide an outlook towards the relevance of these systems for drug delivery and their possible medical application.

## 2. Adamantane Derivatives in Liposomes

Molecular nanotechnology includes different and powerful new tools for understanding biological processes and treatment of human diseases [22]. Multidisciplinary investigations in the fields of chemistry, biology, and medicine contribute to our fundamental knowledge of biomaterials and create novel hybrid materials with practical biomedical applications [23]. Lipids are powerful tools for nanotechnology due to their amphiphilicity and diversity of head and tail chemistry [24]. Liposomes are biodegradable and non-toxic assemblies which can encapsulate both hydrophilic and hydrophobic molecules and are utilized as drug carriers in drug delivery systems [25]. Development of methods for facile control of lipid self-assembly enabled an advancement in the design of new lipid-based drug delivery systems and biomaterials with improved properties [26]. The most effective approach to date is the use of targeted liposomes with surface-attached ligands capable of recognizing and binding to cells of interest, thus increasing liposomal drug accumulation in the desired tissues and organs through passive and active targeting.

Introduction of adamantane into drug molecules often enhances their biological activity and studying the mechanisms of their action broadens our understanding of particular pharmacological profiles. The finding that amantadine is involved in interruption of viral-host fusion in the case of influenza virus and that it inhibits H+ ion channel functions of the viral M2 protein [27] initiated numerous studies of interaction of adamantane compounds with liposomes serving as models for a cell membrane. Several early published papers deal with investigation of partitioning and localization of adamantane and related amantadine within lipid bilayers [21,28,29,30]. Neutron and X-ray studies were undertaken in order to locate amantadine in multilayers of dioleoylphosphatidylcholine (DOPC). The results revealed two populations of amantadine within the bilayer. One site was close to the bilayer surface and the other was much deeper in the hydrophobic core of the bilayer. However, the majority of amantadine occupied the surface site. It was shown that occupancy is dependent upon the initial protonation state of the compound. Under the experimental conditions and with the lipid used no perturbation of bilayer was observed. In order to find out how adamantane compounds are accommodated within a lipid bilayer, other experimental models and different methods were used, including electron paramagnetic resonance (EPR) spectroscopy and molecular dynamic simulations.

Results from recent work in our laboratory—including the studies of adamantyl tripeptides, adamantyl glycopeptides, and adamantyl guanidines, their incorporation into liposomes and interaction with the liposome bilayer as an artificial biological membrane [31,32]—prompted us to review the use of the adamantyl group in designing new targeted drug delivery systems and their application in drug-membrane interaction studies.

### 2.1. Adamantyl Peptides and Adamantyl Glycopeptides in Liposomes

**Figure 1:**
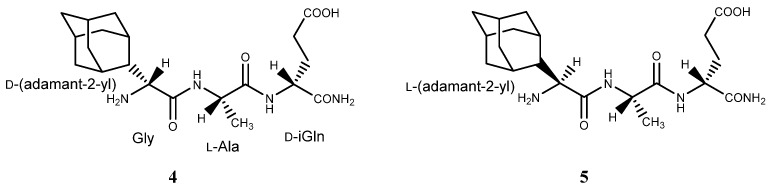
Structures of d- and l-(adamant-2-yl)-l-Ala-d-*iso*Gln (AdTP1 (**4**) and AdTP2 (**5**)).

Adamantyl tripeptides belong to a group of compounds originating from bacterial peptidoglycans [33]. The peptidoglycan fragments of disaccharide pentapeptide were isolated from cultures of penicillin-treated *B. divaricatum*, with a structure consisting of a repeating unit of uncross-linked monomer of the peptidoglycan macromolecule. The disaccharide part is composed of *N*-acetylglucosamine and *N*-acetylmuramic acid, which is a very specific sugar appearing exclusively in bacterial peptidoglycans [34]. Short peptide chains attached to muramic acid via an amide bond through l-alanine also have very characteristic composition of altering l- and d-amino acids. A minimal structure essential for immunostimulating activity is *N*-acetylmuramyl-l-alanyl-d-*iso*glutamine (also called muramyl dipeptide or MDP), which can be recognized as a part of the mentioned peptidoglycan monomer, PGM [35]. It was shown that PGM has versatile biological activity, including antimetastatic and antitumor activity, and that it stimulates immune response in experimental animals [36]. In order to find out how structural modifications of parent PGM molecule affect the biological activity in vivo, adamantyl acetic acid was coupled to PGM [37]. Unfortunately, this molecule showed comparable biological activity to the parent compound. Another line of research was directed toward synthesis of a new type of compounds in which the adamantyl residue was coupled to a short synthetic peptide [38]. The chosen peptide had a composition equivalent to a portion of natural peptidoglycan, l-alanyl-d-*iso*glutamine. The adamantyl moiety was not coupled directly to the peptide, but via a glycine molecule to l-alanine end amino group. Since adamantyl glycine was attached to l-alanine by an amide bond, two diastereoisomers, differing in the configuration of the glycine molecule, were formed (Figure 1). Since the diastereoisomers were separated and characterized, it was possible to study the effect of chirality on biological activity. It was shown that, in most models, both isomers exhibited respective activity, but in some models to a different extent [39].

Interactions of immunostimulating compounds AdTP1 and AdTP2 (Figure 1) with phospholipids in liposomal bilayers were investigated by EPR spectroscopy [40]. The liposomal bilayer was made of egg-phosphatidylcholine (egg-PC), cholesterol, and dicetylphosphate and spin labelled fatty acids (*n*-doxyl stearic acid) with paramagnetic nitroxide moiety positioned at different carbon atoms (namely at 5, 7, and 16 carbon atoms). In the described system, adamantyl tripeptides incorporated in liposomes affected the motional properties of all spin labeled lipids used in the study, even the 16-doxyl stearic acid placed in the hydrophobic core of the liposomes.

In several studies reported earlier, different compounds comprising the elements of a peptidoglycan structure were incorporated into liposomes. Most of these studies concern the synthetically prepared derivatives of muramyldipeptide such as muramyltripeptide phosphatidylethanolamine and 1-adamantylamide-l-alanyl-d-*iso*glutamine [41,42]. The prepared liposomal formulations of the examined compounds were evaluated in vivo and some of them have shown an improved biological activity in the model used. Data about interactions of examined compounds with the lipid bilayer in liposome formulations have not been reported.

**Figure 2:**
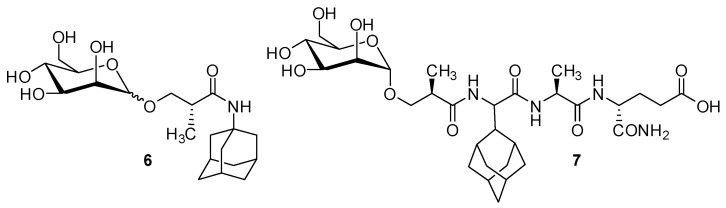
Structures of mannosylated 1-aminoadamantane (**6**) and mannosylated (adaman-2-yl)-tripeptide (**7**).

**Figure 3:**
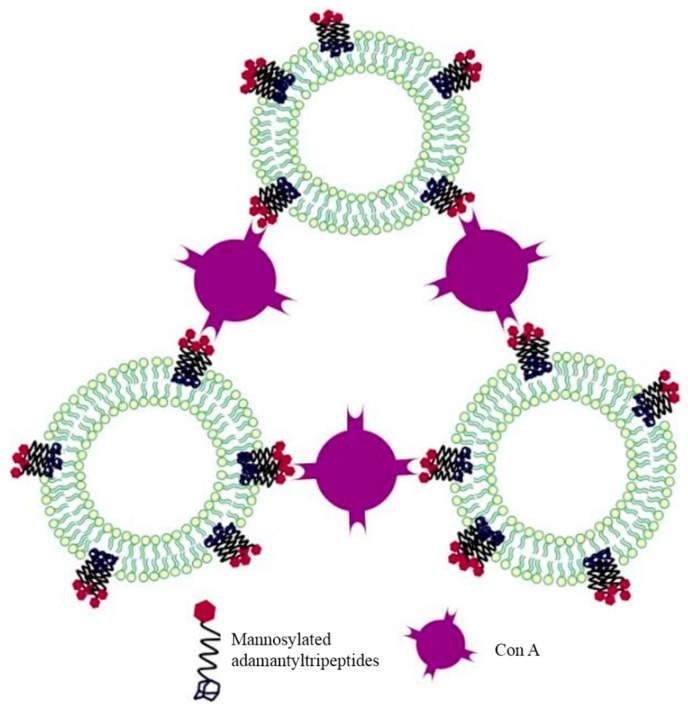
Schematic representation of ConA interaction with mannosylated adamantyl tripeptides anchored via the adamantyl moiety in the lipid bilayer. Reprinted with permission from [31]. Copyright 2012, Elsevier.

In continuation of our interest in the influence of chemical modification of carbohydrates on biological activity, we synthesized mannose derivatives of 1-aminoadamantane and adamant-2-yl-tripeptides (Figure 2). The mannose conjugates of adamantyl tripeptides have been prepared in order to target the mannose receptors present on the cell surface of different immunocompetent cells such as macrophages and dendritic cells, which are considered to be pattern recognition receptors [43,44,45]. Mannosylated adamantyl tripeptides were tested in vivo and their influence on specific immune responses was evaluated. Furthermore, the compounds were incorporated in liposomes and the prepared liposomes were then characterized by using complementary physicochemical methods such as dynamic light scattering (DLS) and atomic force microscopy (AFM) [31]. We showed that the adamantyl moiety, due to its lipophilic properties, was accommodated in the lipid core of the bilayer while the hydrophilic part of the molecule with mannose was exposed on the liposome surface. To confirm this finding we used concanavalin A (ConA), a lectin which specifically binds to the α-d-mannosyl residue. ConA tied the liposomes together and, as a result, an increase in vesicle size and aggregation of liposomes was observed (Figure 3). The described liposomal system with incorporated adamantyl glycoconjugates provided a useful approach to preparation of versatile carbohydrate-decorated targeted drug delivery system where the adamantyl moiety was a membrane anchor for different carbohydrate molecules of interest. In addition, this approach might be a useful model for investigation of specific protein interactions with membrane receptors.

### 2.2. Adamantyl Aminoguanidines in Liposomes

**Figure 4:**
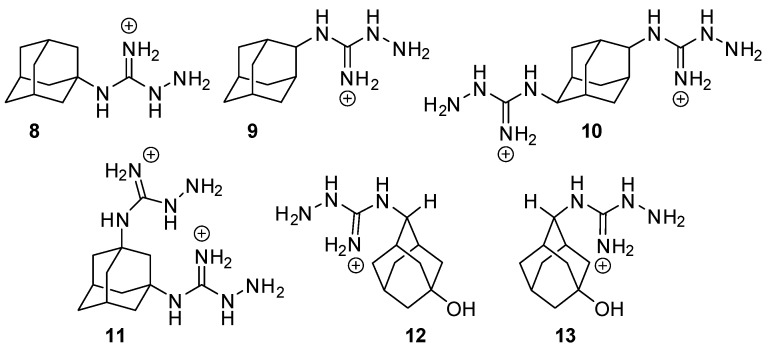
Structures of adamantyl aminoguanidines incorporated into liposomes [32].

We recently reported a successful entrapment of adamantyl aminoguanidine derivatives in liposomes that were acting as model membrane systems [32]. Our goal was to combine, in the same molecule, a highly polar functional group (the guanidine moiety) and a lipophilic anchoring scaffold (the adamantane moiety). The guanidine group represents an important structural motif contained in hydrophilic cell-penetrating peptides. Several studies have shown that interaction of the guanidinium group of arginine, which is part of the examined peptides, with phosphate groups of the membrane’s phospholipids allows their translocation across a cell membrane [46,47]. The prepared adamantyl aminoguanidines (Figure 4) had desirable membrane compatibility features since their adamantyl part served as an effective lipophilic anchor for the lipid bilayer. As the molecules were encapsulated into multilamellar liposomes, the guanidine subunit remained on the outer side of the bilayer pointing outward and thus was enabling the formed vesicle to engage in surface recognition. Supramolecular binding mediated with these guanidine sites is feasible since it is known from the literature that guanidine forms parallel hydrogen bonds with oxoanions [48] and the same is true for the studied adamantyl aminoguanidines [49].

When examining the entrapment efficacy of the studied series of compounds, a correlation between overall lipophilicity of the molecule (expressed as ClogP value) and encapsulation percentage becomes apparent [32]. Although all compounds in the series are constituted of the same key subunits (adamantyl and guanidyl), structural arrangement and spatial distribution of the fragments affects the liposome incorporation. Bis-aminoguanidine compounds are therefore less effective in their incorporation in the lipid bilayer due to an alternating polar-lipophilic character of the molecule. On the other hand, mono-aminoguanidine derivatives possess both a sharply defined charged and a non-polar region and can easily adapt to the requirements of the lipid bilayer. The presence of heteroatoms, in this case hydroxyl groups, also decreases the entrapment efficacy as expected [32].

**Figure 5:**
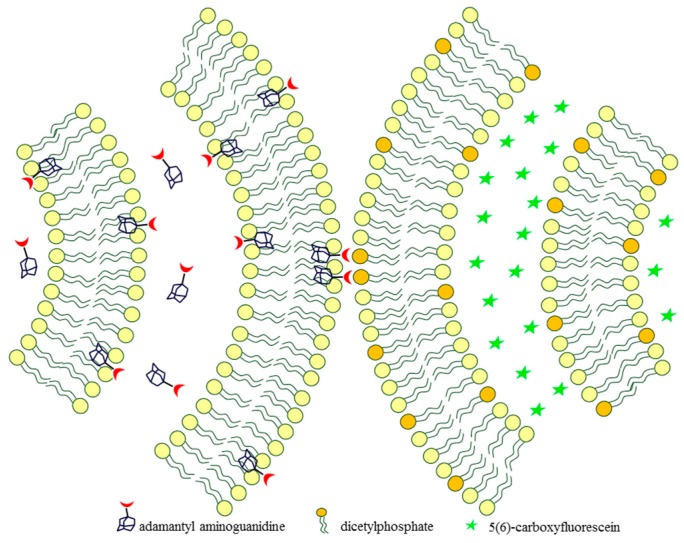
Liposomes containing adamantyl aminoguanidines interacting with complementary liposomes. Reprinted with permission from [32]. Copyright 2014, RSC.

Liposomes containing incorporated adamantyl aminoguanidines were similar in size to empty liposomes, however, surface charge of guest-containing liposomes was significant, further confirming the anchor-recognition site model. The proof-of-principle for molecular recognition action of such encapsulated adamantyl aminoguanidines came from probing liposome interaction. When liposomes containing only phosphate groups and liposomes with studied guest molecules were mixed together, on-surface guanidines successfully interacted with the phosphates of complementary liposomes, resulting in a liposomal recognition and subsequent aggregation event (Figure 5). The resulting multicompartment structures demonstrate membrane fusion processes between vesicles initiated by surface group recognition and are of interest for further intracellular delivery exploration.

## 3. Adamantyl Cyclodextrin Complexes

Nano-devices can also be made of organic polymers, colloids, or biomolecules—including DNA, proteins, and lipids. This alternative approach is known as soft nanotechnology [24]. Special attention was paid to the research of carbohydrates, particularly polysaccharides, and multifunctional characteristics of cyclodextrins (CDs) have enabled them to be used in a variety of drug delivery system, either oral, transdermal, or ocular drug delivery [50]. The commercial viability of CD-based oral formulations has been established with the marketing of more than 20 products worldwide [51,52].

**Figure 6:**
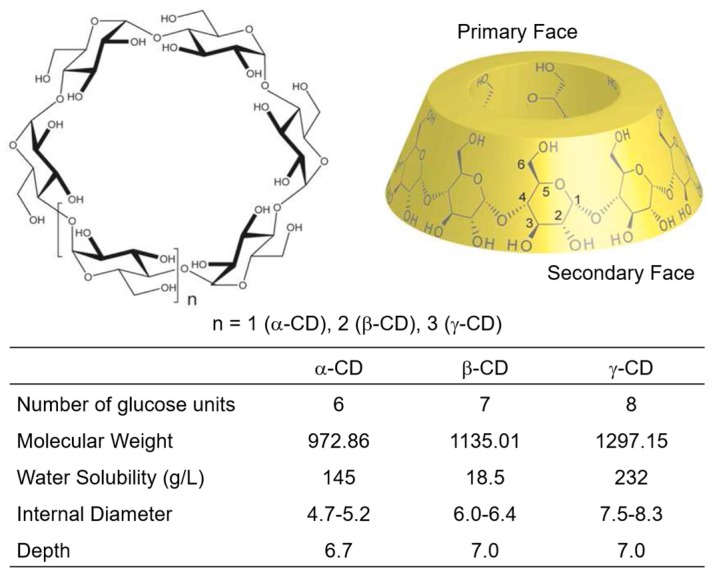
Structures of cyclodextrins (CDs). Reprinted with permission from ref. [66]. Copyright 2008 MDPI.

Cyclodextrins are cyclic α(1-4)oligoglucopyranosides and the cyclodextrin family consists of three well-researched and utilized classes of oligosaccharides, composed of six, seven, or eight glucose units known as α-, β-, and γ-CD (Figure 6) [53]. CDs can be prepared by enzymatic degradation of starch using the enzyme glucosyl transferase (CGTase) [54]. CGTase is produced by many organisms including *Bacillus maverans*. The most stable three-dimensional molecular configuration of CDs takes the form of a hollow truncated cone (torus), which is often also called a doughnut shaped structure. In this structure, the primary hydroxyl groups are orientated at the upper rim of the truncated cone, whereas the secondary hydroxyl groups are orientated at the lower rim. A narrowing of the cone is observed towards the upper rim because of the free rotation of the primary OH group. Due to the presence of hydroxyl groups, the external surface of CD is hydrophilic whereas the cavity containing the glycosidic oxygen is hydrophobic (Figure 6). Because of this hydrophobic nature of the cavity, CDs can serve as host molecules for organic and inorganic compounds (guests). These guest molecules can be included or partly encapsulated without any covalent bond formation. The ability of complex forming can alter the solubility of guest molecules [55] and these inclusion abilities are well-investigated and described in different research areas [56,57]. However, the application of most CDs in pharmaceutics is still restricted since CDs lack cell membrane permeation due to their large size and hydrophilic nature. Additionally, CDs do not bind well enough to drug molecules which are then lost from the CD cavity before they can be delivered to the intended destination. To optimize their applications, natural CDs are usually derivatized [58]. Over the past decade, several groups have reported that amphiphilic CDs can self-assemble to form a variety of aggregates—e.g., bilayers, micelles, and bilayer vesicles [59,60,61]. These aggregates, especially bilayer vesicles, have a potential to overcome the above-mentioned problems. Moreover, these bilayer vesicles could act as drug delivery systems for both hydrophobic and hydrophilic drug molecules. CDs can form inclusion complexes with a wide variety of guest molecules, such as drugs, surfactants, and polymers [23,62,63,64]. This strong interaction of cyclodextrins with different guest molecules is the basis of new drug delivery concepts, based on supramolecular recognition of nanoparticles comprised of an amphiphilic cyclodextrin and a guest molecule. Among various organic groups which are able to interact with cyclodextrins, the adamantyl moiety represents a highly interesting model of a guest since adamantane fits perfectly into the β-cyclodextrin cavity with a high association constant of the order of 10^3^–10^5^ M^−1^ [65].

**Figure 7:**
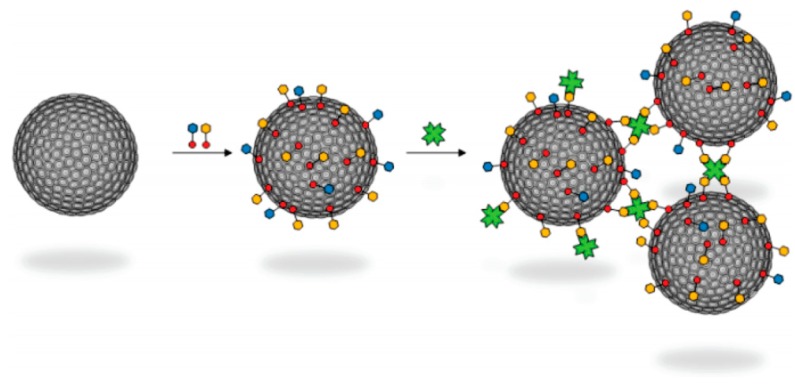
Schematic representation of carbohydrate-decorated cyclodextrin vesicle agglutination induced by the lectin ConA (green) in the presence of a maltose–adamantane conjugate (orange/red). Similarly, agglutination of cyclodextrin vesicles can be induced by PNA (not shown) in the presence of a lactose–adamantane conjugate (blue/red). Reprinted with permission from [72]. Copyright 2011, American Chemical Society.

Taking advantage of this strong interaction and facile host-guest complex formation between adamantane and cyclodextrins, a variety of cyclodextrin-based self-assembled systems for drug and gene delivery—as well as fluorescent sensing and bioimaging—were developed [66,67,68,69]. Furthermore, carbohydrates are recognized as efficient ligands for cellular receptors such as lectins, enhancing molecular transport through biological membranes. It has been shown that adamantylated monosaccharides represent a new class of compounds which can be used for efficient surface decoration of cyclodextrins [70]. Special attention was paid to chemical synthesis of new carbohydrate-adamantane conjugates and studies of their complexes with cyclodextrins as well as interaction with specific lectins. Amphiphilic β-cyclodextrin vesicles decorated with maltose and lactose through host–guest interactions with the adamantane molecule were prepared. The artificial glycocalix on the surface of amphiphilic β-cyclodextrin vesicles was used for studies with specific lectines, concanavalin A (ConA) in the case of maltose and peanut agglutinin (PNA) in the case of lactose (Figure 7) [71,72]. Amphiphilic cyclodextrins were also incorporated into liposomes as artificial receptor units [73].

**Figure 8:**
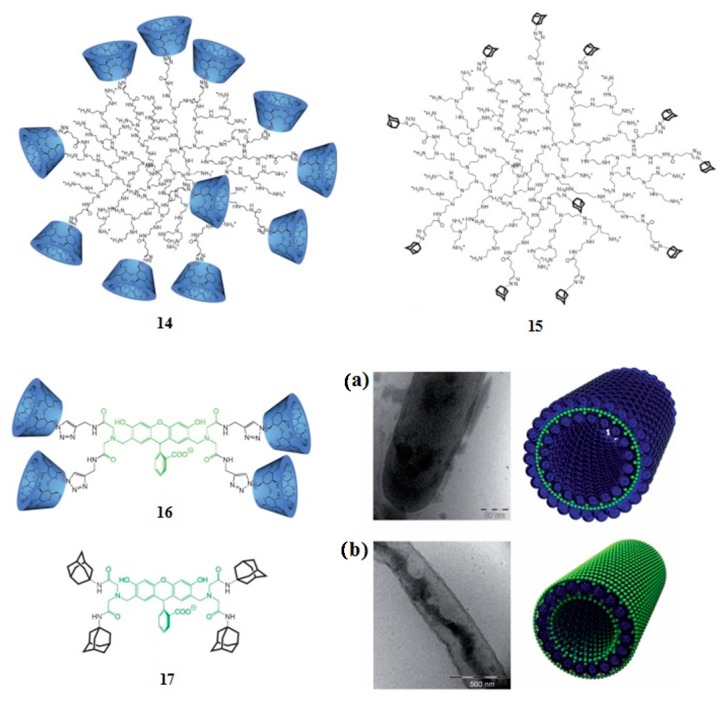
Structures of CD-PEI **14**, Ada-PEI **15**, CD-Cal **16**, and Ada-Cal **17**. (**a**) Cryo-TEM of tubular vesicles prepared from CD-PEI **14** (blue)/Ada-Cal **17** (green); and (**b**) Ada-PEI **15** (blue)/CDCal **16** (green). Reprinted with permission from [74]. Copyright 2011, Wiley-VCH.

Great stability of cyclodextrin-adamantane complexes was utilized in preparation of tubular vesicles of β-cyclodextrin and adamantyl-modified hyperbranched poly(ethylene imine) which were self-assembled with the corresponding modified fluorescent dye calcein (Figure 8) [74]. The structures of tubular vesicles were examined by fluorescence microscopy, cryo-transmission electron microscopy (cryo-TEM), and dynamic light scattering. The tubular structure was confirmed and it was shown that CD-polyethylenimine (CD-PEI) orientates towards the aqueous phase, enclosing the hydrophobic adamantyl fluorescent dye in the interior of the hyperbranched CD-PEI. On the contrary, adamantyl PEI is incorporated into the hydrophilic CD-calcein, giving a double layer of fluorescent dye (Figure 8). The described supramolecular organization had a long-term stability and was stable over a wide range of pHs, providing a great potential for engineering of new materials with improved properties.

## 4. Adamantyl Dendrimers

**Figure 9:**
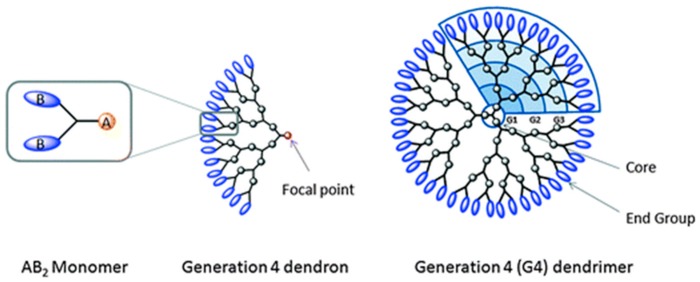
Schematic drawing of an AB_2_ monomer and its subsequent use for the synthesis of a generation four dendron and a generation four dendrimer with a tri-functional core. Reprinted with permission from [76]. Copyright 2012, RSC.

Dendrimers are highly branched macromolecules that display a large number of functional groups on the dendritic framework [75,76]. They are globular polymeric materials with a series of chemical shells built on a core; each shell is called a generation and each branch a dendron (Figure 9). Poly(amidoamine) (PAMAM) dendrimers were the first dendrimer family to be synthesized and characterized [77]. Based on their specific structure and properties, dendrimers are recognized as a unique class of synthetic nanostructures. Dendrimers contain three architectural domains: the multivalent surface, the interior shells surrounding the core, and the core to which dendrons are attached. Dendrimer cores of higher generation of dendrimers, which are protected from the outside by the dendrimer surface, represent special nano-environments that are well-suited for encapsulation of guest molecules. A large number of functional groups on the surface of dendrimers can be further modified and in that way properties of dendrimers can be tuned [78].

**Figure 10:**
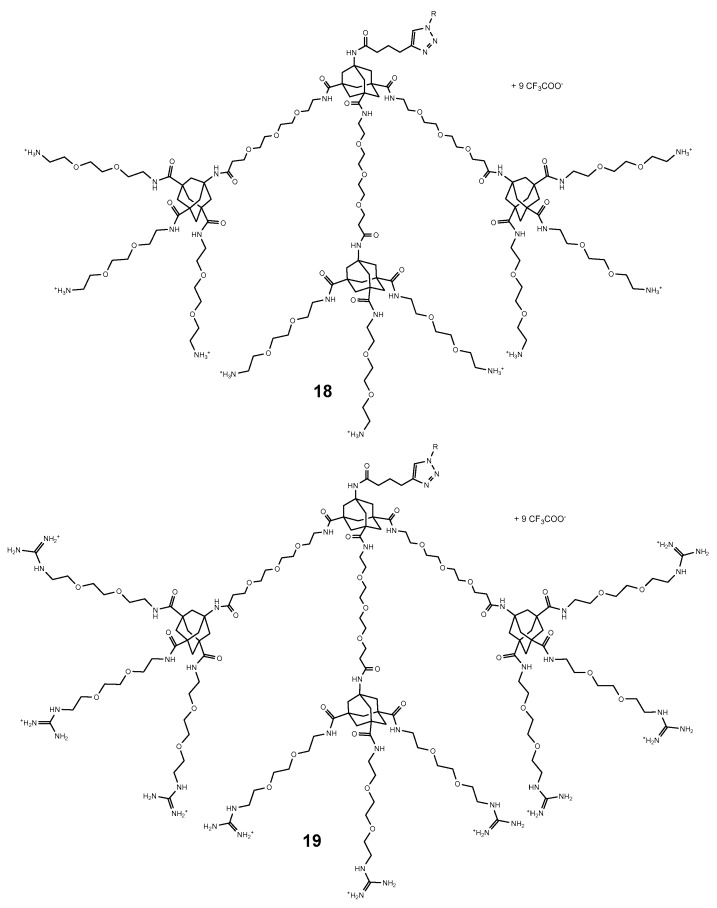
Polycationic adamantane-based dendron (HYDRAmers) with ammonium (**18**) and guanidinium groups (**19**) for complexation with DNA.

Dendrimers containing polyethylenimine (PEI) and polyamidoamine (PAMAM) are the most efficient nonviral transfection agents besides cationic liposomes [79]. They have the ability to successfully deliver genetic material into the cell, but they unfortunately show some undesirable dose-dependent cytotoxicity effects [80]. With the aim of reducing cytotoxicity, a novel type of polycationic dendron was designed and new strategies for the synthesis of different generation Hydra-like dendrons that were based on tetra-functionalized adamantane were developed [81]. Polycationic adamantane-based dendrons of different generations were investigated and preliminary results have confirmed a high cellular uptake of the examined compounds without triggering cytotoxicity [82]. As we mentioned above, chemical and physical properties of dendrimers can be modified by introducing appropriate terminal functional groups as well as by changing the group in the dendrimer core. Adamantane again proves to be an ideal building block since it can form four dendritic arms which are tetrahedrally oriented into space. For example, in adamantyl dendrimers **18** and **19**, peripheral ammonium or guanidinium groups are connected to the adamantane core with short ethylene glycol chains (Figure 10).

In vitro investigations showed that cellular uptake was dependent on the generation of the dendrons used, on the nature of the peripheral groups as well as of the cell type used. Polyammonium and polyguanidinium dendrons were further investigated and used for complexation with plasmid DNA and their transfection activities were evaluated [83]. The size and zeta-potential of the formed hydraplexes were determined using dynamic light scattering (DLS). The optimal N/P ratio, hydramer cationic charge per pDNA negative charge, was determined based on DLS results as well as on agarose gel retardation assay. It was shown that hydramers with periplanar guanidinium groups were able to form smaller and more positively charged hydraplexes compared to the ones with polyammonium dendrons. Additionally, they were able to bring pDNA into the cell and induce gene transfer without triggering significant cytotoxicity effects. There was still a series of parameters that needed to be adjusted for control of hydramer morphology and better pDNA delivery into the cell, but it was undoubtedly demonstrated that polycationic adamantane-based dendrons are promising gene delivery carriers.

Another example of adamantyl-functionalized glycodendrimers taking part in host–guest supramolecular systems with cyclodextrins was described [84]. Modification of a dendrimer surface with carbohydrates greatly diminishes the toxicity of polypropylenimine (PPI) dendrimers and makes them applicable for drug delivery. A new class of hybrid PPI dendrimers of fourth and fifth generation decorated on their surface with adamantyl moieties (hydrophobic guest groups) and maltose or maltotriose units (biocompatible groups) were synthesized. The interactions of hybrid PPI dendrimers with β-CD (host molecules) were studied by NMR and it was shown that the host−guest interaction in the formed supramolecular complexes depends on the number of adamantyl groups on the surface of the glycodendrimer. Further application of adamantane-based dendrons includes their use as carrier systems and scaffolds for multipresentation of bioactive peptides [85,86]. The interaction and binding affinity of conjugated therapeutic P140 peptides on the adamantane-dendron and the target HSPA8 protein was measured by using the surface plasmon resonance (SPR) technique and their activity was tested in vivo as well. It was demonstrated that covalent binding of P140 on adamantane-dendrons does not impair its biological activity. The mentioned examples illustrate a high potential of adamantane-based poly-functional dendritic complex structures for biomedical application.

## 5. Conclusions and Outlook

The unique structural and chemical properties of adamantane provide exceptional opportunities in design of various adamantane-based scaffolds or carrier systems for drug delivery. Adamantane can be used in two ways, as a building block to which different functional groups are covalently bonded (adamantane based dendrimers) or as a part of self-assembled supramolecular systems where the adamantane is accommodated on the basis of its lipophilicity (liposomes) and strong host-guest interaction (cyclodextrins). It should be stressed that adamantane emerged as a suitable scaffold for drug delivery systems because of its biocompatibility and non-toxicity, as well as its low cost and facile accessibility.

Since liposomes are often used as artificial biological membranes, incorporation of adamantane derivatives into these bilayers provides great opportunities in the study of cell recognition due to the possibility of attaching different ligands to the adamantane moiety. As a result, adamantane plays a significant role in understanding interactions of prepared nano vesicles with specific cell receptors and helps illuminate the receptor targeting process in living cells. Preparation and testing of new derivatives of adamantane and other diamondoids therefore remains a highly relevant topic in nanomedicine, especially in the design of safe and selective drug delivery systems and molecular carriers.
